# Case study: patient-derived clear cell adenocarcinoma xenograft model longitudinally predicts treatment response

**DOI:** 10.1038/s41698-018-0060-3

**Published:** 2018-07-11

**Authors:** Roberto Vargas, Priyanka Gopal, Gwendolyn B. Kuzmishin, Robert DeBernardo, Shlomo A. Koyfman, Babal K. Jha, Omar Y. Mian, Jacob Scott, Drew J. Adams, Craig D. Peacock, Mohamed E. Abazeed

**Affiliations:** 1 Gynecologic Oncology Division, Women’s Health Institute, Cleveland Clinic, 9500 Euclid Avenue/A8, Cleveland, OH 44195 USA; 2 Department of Translational Hematology Oncology Research, Cleveland Clinic, 2111 East 96th St/NE6-258, Cleveland, OH 44195 USA; 3 Department of Radiation Oncology, Cleveland Clinic, 9500 Euclid Avenue/CA-60, Cleveland, OH 44195 USA; 4 Department of Genetics, Case Western Reserve University, 2109 Adelbert Road/BRB, Cleveland, OH 44106 USA

## Abstract

There has been little progress in the use of patient-derived xenografts (PDX) to guide individual therapeutic strategies. In part, this can be attributed to the operational challenges of effecting successful engraftment and testing multiple candidate drugs in a clinically workable timeframe. It also remains unclear whether the ancestral tumor will evolve along similar evolutionary trajectories in its human and rodent hosts in response to similar selective pressures (i.e., drugs). Herein, we combine a metastatic clear cell adenocarcinoma PDX with a timely 3 mouse *x* 1 drug experimental design, followed by a co-clinical trial to longitudinally guide a patient’s care. Using this approach, we accurately predict response to first- and second-line therapies in so far as tumor response in mice correlated with the patient’s clinical response to first-line therapy (gemcitabine/nivolumab), development of resistance and response to second-line therapy (paclitaxel/neratinib) before these events were observed in the patient. Treatment resistance to first-line therapy in the PDX is coincident with biologically relevant changes in gene and gene set expression, including upregulation of phase I/II drug metabolism (CYP2C18, UGT2A, and ATP2A1) and DNA interstrand cross-link repair (i.e., XPA, FANCE, FANCG, and FANCL) genes. A total of 5.3% of our engrafted PDX collection is established within 2 weeks of implantation, suggesting our experimental designs can be broadened to other cancers. These findings could have significant implications for PDX-based avatars of aggressive human cancers.

## Introduction

There is an urgent need for models of human cancer that can reliably predict clinical activity. Patient-derived xenograft (PDX) models can faithfully recapitulate their tumors of origin by several important biological criteria and may predict patient drug response.^1–3^ However, with engraftment times of several months for most cancer types and additional intervals of time required for graft expansion and drug testing, it remains unclear whether these models can be used to inform individual therapeutic strategies in a clinically acceptable timeframe.^4,5^ If successful, strategies to improve the efficiency and speed of engraftment, including appropriate cancer type selection, can accelerate the adoption of PDXs for clinical prediction.

Beyond the practical obstacles of developing PDX models, their fidelity to the tumor in its original host have not been thoroughly examined longitudinally. Evolutionary dynamics is predicted to confer flux in the tumor genetic landscape and this dynamism could represent a significant obstacle to prediction.^6^ The extent that initial and collateral drug responses co-evolve in the PDX and patient remain largely unknown. Studies to date have either largely assumed temporal fixation in drug sensitivity or do not account for the sequencing of drugs. These approaches are likely to be prone to inaccuracies in clinical prediction due to changes in tumoral landscapes under selection.^7–9^

Clear cell adenocarcinoma of müllerian origin (cervix, endometrium, and fallopian tubes) are aggressive tumors with a propensity for rapid dissemination.^10^ Due to the few number of women affected per year this cancer type has been difficult to study clinically, precluding a consensus on management. Estimates of outcomes for patients with advanced stages of disease are dismal, with an estimated 24-month overall survival of <15%.^11^ However, and in part attributed to its aggressiveness, there are several important features that nominate personalized avatar studies in clear cell adenocarcinomas. These include ample donor tissue owing to frequent upfront surgery, rapid intrinsic tumor proliferation rates and the absence of a definitive standard of care. The lack, or even prospect, of an established standard of care means that reliable models can add substantial clinical value.^12^ Against this background, we established a PDX from a patient with metastatic clear cell adenocarcinoma and developed 3*x*1 (three mice per drug) randomization and co-clinical trial designs to correlate avatar-directed predictions with clinical outcomes.

## Results

### Case history

**Fig. 1:**
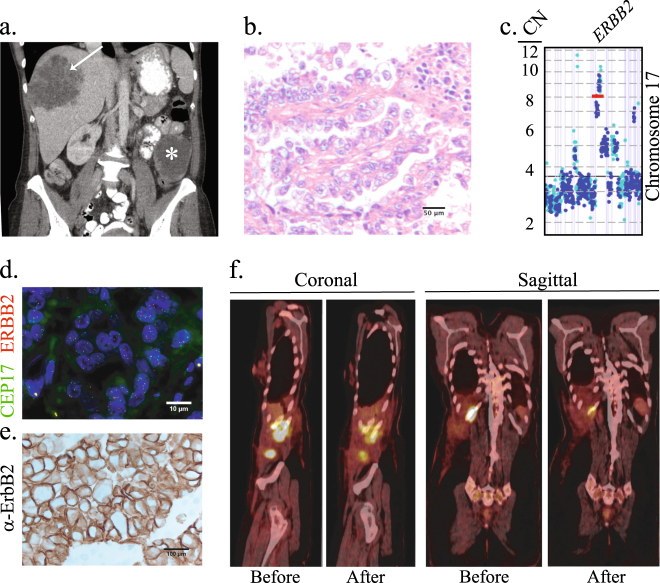
Clinical course and molecular profiling of the primary tumor. **a** Computed tomography scan obtained at the time of diagnosis. The 8.2 × 8.8 cm lobulated metastatic mass in the liver (arrow) and the 8.0 cm centrally necrotic primary tumor mass in the left mesentery (asterisk) are shown. **b** Representative image of an H&E stained section of the primary tumor. **c** Copy number count estimates from both exonic (blue) and intragenic or intronic (cyan) reads in Chromosome 17 are shown. **d** Representative FISH image of the primary tumor using ERBB2/CEP17 dual-color probes. The average *ERBB2* signal copy number was 5.1 and the *ERBB2*/*CEP17* ratio was 2.0. **e** Representative ErbB2 IHC image of the primary tumor. **f** Sagittal and coronal images of the PET/CT scans before and after treatment with three cycles of paclitaxel and neratinib (second-line treatment). The SUV maximum value for each lesion before and after treatment was, respectively: right lateral abdominal wall musculature, 11.7 and 6.6; posterior 11th rib, 11.2 and 4.1; and the soft tissue abutting the hepatic surgical site, 14.8 and 7.9.

A 49-year-old woman with a history of endometriosis presented with left flank pain and acute blood loss. A computed tomography (CT) scan demonstrated a large mass in the posterior segment of the right hepatic lobe and a centrally necrotic mass in the left mesentery (Fig. 1a). A biopsy of the liver mass demonstrated clear cell adenocarcinoma, müllerian type. She underwent staged surgical management consisting of omentectomy, modified radical hysterectomy, bilateral salpingo-oopherectomy and bilateral pelvic and para-aortic lymph node dissection followed by right hepatic metastectomy with diaphragmatic resection. Pathology confirmed a diagnosis of clear cell adenocarcinoma, müllerian type in the omentum and liver but not in the uterus or ovaries. The histologic section demonstrated an epithelial neoplasm with papillary architecture, composed of pleomorphic and hyperchromatic tumor cells with cleared to pink cytoplasm (Fig. 1b). The morphology, in addition to tumor cells staining positive for CK7/PAX8 and negative for ER/WT-1 (not shown), was consistent with the diagnosis of clear cell adenocarcinoma.^13^ Whole exome sequencing of the primary tumor sample revealed *ERBB2* amplification (Fig. 1c), which was confirmed by FISH (Fig. 1d). ErbB2 IHC demonstrated homogenous, dark circumferential staining in greater than 10% of tumor cells, consistent with a score of 3+ (Fig. 1e).

Re-staging CT scans after surgery revealed new right inguinal lymphadenopathy, an enlarging right chest wall mass (separate from area of resection) and an abdominal incisional recurrence, indicating widely metastatic disease. She received first-line gemcitabine (1000 mg/m^2^ [d1, d8] every 3 weeks) and nivolumab (3 mg/kg [d1, d15] monthly) 4 weeks after the hepatic metastectomy. Re-staging CT scans after 5 cycles of therapy (5 months) demonstrated a partial response in all known sites of disease and no evidence of new lesions. However, shortly thereafter she developed progressive pain. Re-staging PET/CT and MRI brain scans demonstrated progression of disease in the perihepatic region and the right chest wall, new tumor implants along the right lateral abdominal wall musculature, posterior 11th rib, anterior 10th rib, and a new solitary brain metastasis. She underwent craniotomy and brain metastectomy and stereotactic radiosurgery to the surgical bed. After recovering from surgery, she was treated with paclitaxel (80 mg/m^2^ every 3 weeks) and neratinib (160 mg daily) for 4 cycles followed by neratinib alone. A re-staging PET/CT and brain CT after 4 cycles of therapy revealed no evidence of new disease and a partial metabolic response in the areas of known disease (Fig. 1f). Consolidative radiotherapy and cryoablation were delivered to areas of minimal residual disease in the chest wall. Her disease burden at 24 months from diagnosis consisted of a solitary focus of disease in the chest wall that was partially metabolically active, with plans for additional local therapy followed by discontinuation of systemic treatment for a period of observation.

### Model development, growth kinetics, and genetic fidelity

**Fig. 2:**
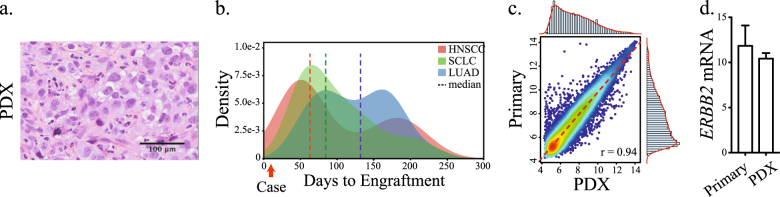
Time to engraftment and comparison of genome-wide gene expression profiles. **a** Representative image of an H&E stained section of the PDX. The histologic section is composed of pleomorphic and hyperchromatic tumor cells with cleared to pink cytoplasms, similar to the tumor of origin. The papillary architecture and hyalinized stroma is present but not as prominent as in Fig. 1b due to the cellular density of the PDX. **b** Probability density function of time to engraftment for 36 small cell lung carcinoma PDXs (*SCLC*), 27 head and neck squamous cell carcinomas (*HNSCC*) and 27 lung adenocarcinoma PDXs (*LUAD*). Red arrow represents the time to engraftment of the index case. Samples obtained from pleural effusion were excluded from this analysis. **c** Scatter plot, linear regression (dashed red line) and probability density histograms of genome-wide gene transcript levels (16,599 transcripts) in the donor tumor and the PDX. Pearson’s *r* correlation coefficient is shown. **d** *ERBB2* mRNA levels in the donor tumor and PDX. Data are expressed as the means ± s.e.

Fresh tissue obtained from the liver metastectomy was processed and implanted into the flank of an NSG mouse several hours after the patient’s initial surgery. Within 10 days, a palpable tumor, with histopathologic features consistent with clear cell adenocarcinoma, was noted at the site of injection (Fig. 2a). Figure 2b demonstrates the time to engraftment of the index case juxtaposed with cohorts from rapidly proliferating tumors (i.e., small cell lung carcinoma and squamous cell carcinomas) and tumors with lower proliferation rates overall (i.e., lung adenocarcinoma). There was concordance between the average time to engraftment and the clinically observed doubling time for each histologic subtype, providing additional evidence of the phenotypic similarity between PDXs and their tumors of origin.^14^ In our inventory of >220 PDXs derived from multiple cancer types, 5.3% have developed within 2 weeks of implantation, suggesting that rapid engraftments occur at a low but not insignificant rate.

The matched donor tumor and the PDX were subjected to genome-wide gene expression profiling, which demonstrated high transcriptomic concordance (Pearson *r* = 0.94) (Fig. 2c). There were no differentially expressed genes between the primary tumor and the PDX (Benjamini-Hochberg adjusted *P*-value threshold = 0.05). *ERBB2* gene amplification was identified in the PDX (copy number estimate of 7.9) and the levels of *ERBB2* mRNA in the PDX and the primary tumor were similar (Fig. 2d). These data indicate that there was high genomic fidelity between the PDX and the donor tumor from which it was derived.

### First-line responses and treatment resistance

**Fig. 3:**
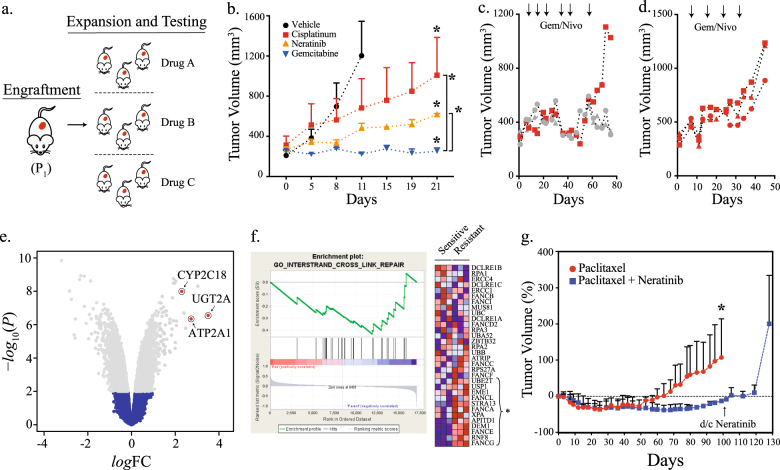
Drug efficacy studies in PDX and treatment resistance. **a** Schematic of the 3*x*1 design and treatment arms. **b** NSG female mice bearing PDX were block randomized into one of four treatment arms as shown. Data are expressed as the means ± s.e. *P*-values of the *χ*^2^-test between vehicle and the treatment groups, cisplatinum, neratinib, and gemcitabine, were 0.03, 0.001, and <0.001, respectively. *P*-values between the treatment groups cisplatinum/neratinib, cisplatinum/gemcitabine, and neratinib/gemcitabine treatment groups were 0.06, 0.003, and <0.001, respectively. **c** NSG mice bearing PDX were treated with gemcitabine and nivolumab until treatment resistance was established. **d** Tumor from the secondarily resistant mouse in **c** were allowed to grow to ~400-500 mm^3^ and re-challenged with gemcitabine and nivolumab. **e** Differential gene expression of equally passaged (P_4_) untreated and treatment-resistant PDX. The gray dots represent differentially expressed gene based on *P* < 0.01 and FDR = 2.5%. **f** GSEA analysis of treatment-resistant PDX. Gene expression heatmap of genes within the GO Interstand Cross Link Repair pathway for each biological replicate is shown. **g** PDX from post-gemcitabine/nivolumab treated mice were block randomized into two treatment arms as shown. Neratinib was discontinued at ~100 days from the time of randomization in the paclitaxel plus neratinib arm. Data are expressed as the means ± s.e. The *p*-value of the *χ*^2^-test was 0.003.

After initial engraftment (P_1_), the PDX was passaged by single-step propagation into 12 mice representing four cohorts that received treatment in parallel with the indicated regimens (Fig. 3a). The drugs tested were selected based on deliberations of a multidisciplinary tumor board. Due to coincident PDL-1 amplification and expression, the multidisciplinary tumor recommended the combination of nivolumab with either cisplatinum, gemcitabine or, in light of evidence of *ERBB2* amplification, an anti-ErbB2 agent. Cisplatinum-based regimens represent first-line therapies for patients with clear cell adenocarcinoma of the endometrium. Our results indicated that this particular PDX was marginally responsive to cisplatinum (Fig. 3b). A comparison of all three drugs indicated that gemcitabine was the most effective at preventing tumor growth.

The patient ultimately received the combination of gemcitabine and nivolumab. To assess longitudinal responses and monitor for disease progression, the PDX was subjected to rounds of gemcitabine and nivolumab. During the third round of nivolumab and gemcitabine, progression, despite therapy, was noted in 1 of the 3 mice (Fig. 3c). The putatively resistant tumor was re-implanted into three additional mice and challenged once again with gemcitabine and nivolumab. Tumors grew shortly after drug re-challenge, confirming treatment resistance (Fig. 3d). Tumor tissue from the treatment-resistant PDX were subjected to genome-wide gene expression profiling. Differential gene expression analysis between equally passaged (P_4_) and treatment-resistant tumors revealed upregulation of genes critical for drug metabolism and detoxification (*CYP2C18*, *UGT2A*, and *ATP2A1*) (Fig. 3e and Table S1) and GSEA revealed enrichment for pathways implicated in DNA interstrand cross-link repair (ES = −0.433; *P* = 0.012; FDR = 0.28) (Fig. 3f). Driving this association were significant increases in the expression of genes including *XPA*, *FANCE*, *FANCG*, and *FANCL*. These results indicate that upregulation of biological pathways previously implicated in drug (single-gene) and gemcitabine (gene set) resistance were associated with onset of therapeutic resistance in the PDX.^15^

### Longitudinal prediction

In anticipation of resistance in the patient and to predict the most optimal second-line therapy, the treatment-resistant PDXs were again randomized on the basis of deliberations by the multidisciplinary tumor board. Mice either received paclitaxel alone or paclitaxel and neratinib followed by neratinib. The combination of paclitaxel and neratinib demonstrated greater tumor control than paclitaxel alone (Fig. 3g). To further assess the activity of neratinib, its administration was discontinued at ~100 days. Shortly after discontinuation, tumors rapidly increased in volume, indicating a potent growth inhibitory effect by neratinib in the adjuvant setting. In line with these predictions, the patient responded to second-line paclitaxel with neratinib delivered concurrently and adjuvantly (Fig. 1f).

## Discussion

**Fig. 4:**
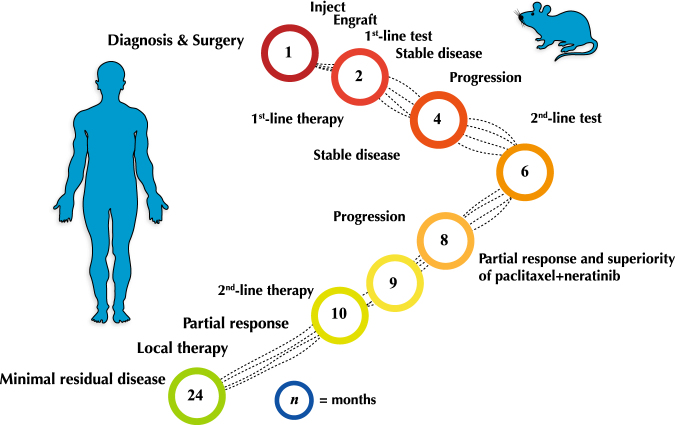
Accelerated and concordant outcomes in mice. A timeline of events in the mouse and patient

To determine whether a cancer model can presage clinical outcomes, we developed a PDX from a patient with metastatic clear cell adenocarcinoma and used a murine 3*x*1 and co-clinical experimental design to effectively guide a patient’s clinical management. We showed that the use of a genetically concordant experimental model has the potential to improve response rates in a cancer type that confers a poor prognosis and is frequently treatment refractory.^10^ We also showed that tumor response in mice correlated with the patient’s clinical outcomes beyond first-line therapy. We predicted both the development of resistance and response to second-line therapy before these events were observed in the patient (Fig. 4). We showed gene expression changes in the resistant PDX concurred with biologically plausible mechanisms of therapeutic resistance. Namely, phase I/II drug metabolism enzymes (*CYP2C18*, *UGT2A*, and *ATP2A1*) and genes implicated in DNA interstrand cross-link repair (e.g., *XPA*, *FANCE*, *FANCG*, and *FANCL*) were significantly upregulated in the treatment-resistant PDX. Both of these pathways have been previously implicated in conferring resistance to several classes of chemotherapeutics,^16,17^ including gemcitabine.^15,18^

The practice of using PDX to guide treatment decisions can be stymied by implant geographic stratification bias, genetic divergence and slow engraftment rates.^19^ Despite this, previous work has suggested that the approach is possible and can potentially correlate with patient responses if these obstacles can be overcome.^20^ Our experimental design was aided by several factors that permitted prospective treatment response projections. The availability of a large quantity of tissue from a surgical specimen allowed for multi-region tumor sampling for PDX generation, which likely contributed to genetic fidelity. In addition, the intrinsic rapid proliferation rate of the patient’s clear cell adenocarcinoma and the requisite recovery time after surgery allowed us to share our experimental results with a multidisciplinary tumor board within a clinically acceptable schedule.

The ability to test several drugs in a timely manner was also facilitated by our statistical methodology. After initial engraftment, the harvested P_1_ tumor is used to generate P_2_ mice with the competing goals of achieving both a sufficient sample size and a rapid secondary engraftment. The latter is attributed to the relationship between the volume of tumor tissue injected into individual mice and the time to engraftment. An accommodation between the number of mice per treatment group and the total number of treatment groups (i.e., the number of drugs that can be tested) was necessary. We used a regression model with an improved efficiency of testing to maximize our statistical power.^21^ The model achieves this efficiency by assessing intra-animal correlation patterns, uses the entire data series and, critically, does not execute a test at each time point, which can inflate the type I error rate. Therefore, our statistical design can achieve optimal power with a minimal sample size, allowing for both accurate and timely testing.

Although only 5.3% of our successfully generated PDX were of suitable size to be harvested within 2 weeks of implantation, a narrower focus on rapidly proliferating tumors is expected to increase the proportion of patients who may be eligible for similar studies. Our models are currently limited in that they may not fully assess the efficacy of immunotherapies. However, ongoing efforts to develop autologous immune-humanized PDX models are poised to improve their predictive potential.^22^ Altogether, and despite this limitation, our results establish the feasibility of our pre- and co-clinical trial design and its potential for implementation in similar settings.

The drug regimens that we tested in the mice were in part informed by genomic analyses (e.g., *ERBB2* amplification and anti-ErbB2 therapy), demonstrating the successful integration of PDXs with other personalized medicine approaches. Such genotype-phenotype correlations derived from an *n* of one may prove powerful if findings can be extrapolated to other patients with the same cancer type and genetic alteration. For example, the frequent amplification of *ERBB2* in clear cell adenocarcinoma of müllerian origin suggests that the favorable results we observed with a small molecule tyrosine kinase inhibitors of ErbB2 could be extended to other patients.^23^ Finally, we note that our patient is alive with minimal residual disease 24 months from the time of diagnosis. This is well beyond the median survival estimate of ~3 months for patients with a similar stage of disease.^24^ Although care must be taken to avoid generalizing the favorable clinical outcome observed in our patient, our results overall indicate that more studies exploring PDX-directed therapies in aggressive cancers are warranted.

## Methods

### Mouse xenograft studies

NSG mice were obtained from the Cleveland Clinic Biological Resources Unit facility. All mouse studies were conducted under a protocol approved by the Cleveland Clinic Institutional Animal Care and Use Committee. The sample collection protocol was approved by the Institutional Review Board at the Cleveland Clinic and biological material was obtained from the patient who provided written informed consent. Tumors were mechanically processed into sub-millimeter pieces in antibiotic-containing RPMI medium, combined with Matrigel and implanted into the flank of a 6- to 8-week-old female NSG mice using syringe with a 20G needle. Tumors were harvested and stored for biological assays on reaching a size of >1000 mm^3^. Mice were randomized when tumors reached ~200–300 mm^3^ in volume. Three mice were randomized into each treatment group (3*x*1). Drugs were formulated according to the manufacturer’s specifications. Gemcitabine (240 mg/kg), paclitaxel (20 mg/kg), and cisplatinum (5 mg/kg) were administered by weekly intraperitoneal injections for three consecutive weeks, representing a “round” of therapy. Neratinib (40 mg/kg) was administered daily by oral gavage. Mice used for longitudinal treatments with first-line therapy (gemcitabine and nivolumab) were primed with peripheral blood lymphocytes (PBLs) isolated from the patients’ blood. PBLs were injected into the tail vein 48 h prior to drug treatments. The mice received nivolumab (200 μg per mouse) and gemcitabine (240 mg/kg) by intraperitoneal injections until resistance (growth despite treatment) was observed. Drug dosage was guided by previously established therapeutic levels in cell line xenograft models^25–28^ and the maximum tolerated doses in mice.^29^ If appropriate, adjustments to an individual treatment dose was made based on our observations of efficacy and toxicity with the specific formulations in NSG mice. Tumor volume was calculated using the formula: (length × width^2^)/2.

### Genetic and molecular profiling

Genomic DNA was extracted according to QIAamp DNA mini-Kit protocols (Qiagen). Whole-exome capture was accomplished based on liquid phase hybridization of sonicated genomic DNA having 150−200 bp mean length to the bait cRNA library synthesized on magnetic beads (SureSelect, ver. 3 or 4 (Agilent Technology), according to the manufacturer’s protocol. The captured targets were subjected to massively parallel sequencing using the HiSeq 2000 with the paired-end 75–108 bp read option, according to the manufacturer’s instructions. Copy number analysis was derived alongside a process-matched normal control (an internally validated mixture of 10 heterozygous diploid HAPMAP control samples) in order to normalize a sample’s sequence coverage distribution across baited targets as previously described.^30^ Briefly, normalized coverage data for exonic, intronic, and SNP targets accounting for stromal admixture are plotted on a logarithmic scale and minor allele SNP frequencies are concordantly plotted across the genome. Cluster groupings of targets and minor allele SNPs are further used to define upper and lower bounds of genomic segments. Empirical Bayesian algorithms employ a distribution of parameters including purity and base ploidy and probability matrices are derived to fit these data and generate copy number alteration variant calls. Since each copy number model is dynamically generated for individual samples, confidence intervals vary with sample data but achieved high performance (sensitivity was 99% with PPV >99%) within a range of 20–75% tumor content. Gene amplification was manually evaluated on tissue by interphase fluorescence in situ hybridization (FISH), using a dual label probe set for the ERBB2 locus and the alphacentromeric region of chromosome 17.

Total RNA was extracted from tumor samples using a TRIzol method (Invitrogen), purified with an RNeasy kit (Qiagen). RNA samples were profiled using the HumanHT-12 v4 Expression BeadChip array (Illumina) in at least duplicates. Data were processed using GenomeStudio version 2011.1 and the limma package.^31^

Tissue derived from the primary tumor and PDX were stained with Haematoxylin-Eosin-Saffron (HES) and immunohistochemical staining was performed using the anti-Her2 polyclonal antibody clone 4B5 (Ventana, Tuscon, AZ).

### Statistical analysis

Statistical analysis was performed using R 3.4.1 (R Foundation for Statistical Computing, Vienna, Austria).^32^ To establish whether PDX intergroup differences were significant, we used regression with random effect and autoregressive errors (RE/AR).^21^ The error term in this model is the sum of the random effect and autoregression. The random effect measures the random difference between each animal and the mean volume for all animals. Autoregression measures the random process in which the correlation between observations decreases with increasing separation in time. Altogether, the model is multivariate, in that it treats the tumor volume series of an individual animal as a single-multivariate observation, uses the entire data series of volume measurements and measures intra-animal correlation patterns. The key assumptions of the model include: independence, symmetry, same variance in all groups, that correlations follow the RE/AR model and that the growth curve can be adequately represented by a parametrized equation. The assumption of the model were assessed for individual groups and power transformation (log) of volume measurements was used to fulfill the requisite assumptions when indicated. Using RE/AR, the type I error and power estimates for the sample size in our 3*x*1 experimental design (*n* = 3) were <0.05 and >90%. A likelihood ratio test was used to compare the null model (assumes same slope in each group) to the alternative model (assumes different slopes in each group) to assess differences between treatment groups. A *p*-value associated to the *χ*^2^-test of <0.05 was considered to be statistically significant and was calculated using the R package *agce*.^33^ For differential gene expression analyses, the log2 fold-change and adjusted *p*-values (using the Benjamini-Hochberg procedure) were calculated using linear models in combination with the moderated *t*-statistic using the R package *limma*.^34^

### Data availability

The datasets generated and/or analyzed during the current study are either included in this published article or available in the GEO repository: https://www.ncbi.nlm.nih.gov/geo/query/acc.cgi?acc=GSE115455.

## Electronic supplementary material

Table (SI)

## Electronic supplementary material

**Supplementary information** accompanies the paper on the *npj Precision Oncology* website (10.1038/s41698-018-0060-3).
